# *PRTN3* variant correlates with increased autoantigen levels and relapse risk in PR3-ANCA versus MPO-ANCA disease

**DOI:** 10.1172/jci.insight.166107

**Published:** 2023-02-22

**Authors:** Dhruti P. Chen, Claudia P. Aiello, DeMoris McCoy, Taylor Stamey, Jiajin Yang, Susan L. Hogan, Yichun Hu, Vimal K. Derebail, Eveline Y. Wu, J. Charles Jennette, Ronald J. Falk, Dominic J. Ciavatta

**Affiliations:** 1 University of North Carolina Kidney Center, Division of Nephrology and Hypertension, Department of Medicine, University of North Carolina at Chapel Hill, Chapel Hill, North Carolina, USA.; 2 Division of Pediatric Allergy, Immunology, and Rheumatology, Department of Pediatrics, University of North Carolina, Chapel Hill, North Carolina, USA.; 3 Department of Pathology and Laboratory Medicine, and; 4 Department of Genetics, University of North Carolina at Chapel Hill, Chapel Hill, North Carolina, USA.

**Keywords:** Autoimmunity, Autoimmune diseases, Vasculitis

## Abstract

A GWAS of patients with anti-neutrophil cytoplasmic antibodies (ANCAs) found an association between proteinase-3 ANCA (PR3-ANCA) and a single nucleotide polymorphism (rs62132293) upstream of *PRTN3*, encoding PR3. The variant (G allele) was shown to be an expression quantitative trait locus in healthy controls, but the clinical impact remains unknown. Longitudinally followed patients with ANCA and healthy controls were genotyped. Gene expression was quantified by real-time quantitative PCR from leukocyte RNA. Plasma PR3 was quantified by ELISA. Among patients, variant carriers had elevated leukocyte *PRTN3* expression compared with noncarriers (C/G vs. C/C and G/G vs. C/C). Healthy controls had low *PRTN3* regardless of genotype. Myeloperoxidase (*MPO*) expression did not differ by genotype. *PRTN3* expression correlated with circulating PR3, and variant carriers had higher plasma PR3 compared with noncarriers. Among variant carriers, there was an increased risk of relapse in patients with PR3-ANCA versus MPO-ANCA. The risk allele marked by rs62132293 is clinically significant as it is associated with increased autoantigen and may, in part, explain increased relapse in PR3-ANCA. Our results underscore the role of autoantigen availability in ANCA vasculitis.

## Introduction

GWAS have identified genetic variants that predispose individuals to anti-neutrophil cytoplasmic antibody (ANCA) vasculitis. The ANCA target, proteinase-3 (PR3), is a neutrophil-derived protease encoded by *PRTN3*, and genetic variants at the *PRTN3* locus have been identified in association with granulomatosis with polyangitis (GPA)/PR3-ANCA (1–4). A single nucleotide polymorphism (SNP), rs62132293, found upstream of the *PRTN3* transcription start site, was shown to be an expression quantitative trait locus (eQTL) for *PRTN3* expression (message) in healthy controls (2). While there is no direct evidence yet of increased circulating autoantigen (PR3) due to rs62132293, a linked SNP was identified as a protein quantitative trait locus in a large proteomic study (5). The relevance of this SNP and autoantigen availability in patients with ANCA vasculitis remains unknown.

It has been shown that patients with ANCA vasculitis have increased circulating PR3 compared with healthy individuals or disease controls, suggesting a pathogenic role of the autoantigen (6, 7). Studies have reported increased transcription results in newly synthesized autoantigens from patient neutrophils (8). Furthermore, animal studies show increased autoantigen expression is associated with more severe ANCA vasculitis (9, 10). Taken together, *PRTN3* gene expression (message) elevations impact circulating autoantigen production that could predispose patients to more severe disease. rs62132293 is a known eQTL for *PRTN3* gene expression in healthy individuals, but the influence of the variant on gene expression, circulating PR3, and clinical impact among patients has not been previously explored to our knowledge.

We hypothesized that rs62132293 has a functional role in disease by increasing autoantigen (PR3) availability. We postulated that rs62132293 is linked to increased target autoantigen gene expression (message), which translates to autoantigen availability (protein) and a more severe clinical phenotype in the setting of corresponding ANCAs (PR3). Clinicians have long associated PR3-ANCA with increased relapse risk compared with myeloperoxidase ANCA (MPO-ANCA) (11). We show that this risk may, in part, be explained by increased PR3 availability.

## Results

We genotyped patients and healthy individuals and found the C/C genotype (noncarrier) in 179 (44%) and 62 (48%), C/G genotype (heterozygous carrier) in 181 (45%) and 54 (41%), and G/G genotype (homozygous carrier) in 41 (10%) and 14 (11%) of patients and healthy controls, respectively. Patient characteristics are shown in Table 1. Our cohort consisted of the following serotypes: 197 (49%) with MPO-ANCA, 170 (42%) with PR3-ANCA, 9 (2%) who were dual positive for MPO and PR3-ANCA, and 25 (6%) who were seronegative. GPA was diagnosed in 147 (37%); microscopic polyangitis was diagnosed in 184 (46%); and the remainder of patients had renally limited disease (*n* = 53, 13%), eosinophilic GPA (*n* = 9, 2%), or missing definitive diagnosis (*n* = 8, 2%).

In the patient cohort with MPO-ANCA or PR3-ANCA (*n* = 376), patients with PR3-ANCA were more likely to carry the G allele than MPO-ANCA patients (OR = 1.46, 95% CI: 1.07, 2.00, *P* = 0.0164). The frequency of patients with PR3-ANCA who were homozygous for the variant was greater than the frequency of patients with MPO-ANCA (20/170, 11.8% versus 15/197, 7.6%). Homozygous (G/G) carriers were younger at onset of disease (median age 42 years), compared with noncarriers (C/C; 56 years, *P* = 0.006) and heterozygotes (C/G; 54 years, *P* = 0.01). There was no difference in the cohort composition with respect to pediatric patients (Table 1). In a serospecific sensitivity analysis, the difference in age at onset persisted in PR3-ANCA but not in MPO-ANCA (Supplemental Tables 2 and 3; supplemental material available online with this article; https://doi.org/10.1172/jci.insight.166107DS1). Genotype groups did not differ in race/sex, organ involvement, or estimated glomerular filtration rate at disease onset. The predominant organ involvement at onset was renal disease and similar between groups.

### Homozygous (G/G) and heterozygous (C/G) genotypes are associated with higher leukocyte PRTN3 but not MPO gene expression.

**Figure 1:**
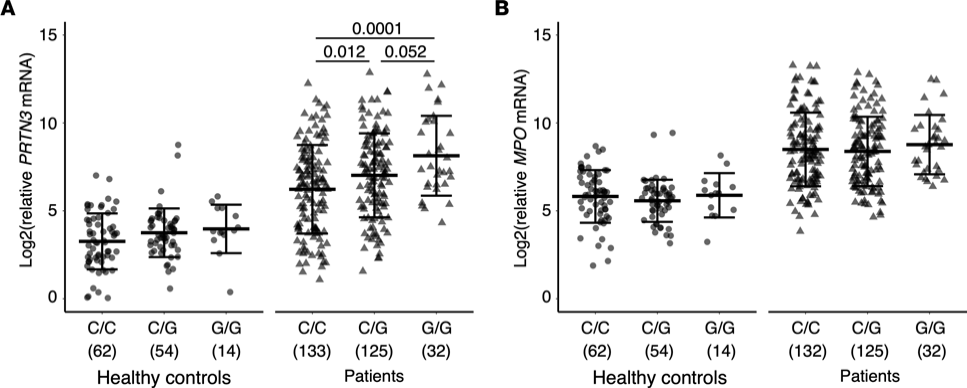
Autoantigen gene expression in total leukocytes. (**A**) *PRTN3* and (**B**) *MPO* gene expression in healthy controls (circles) and patients (triangles). Healthy control and patient samples were separated by genotype for *PRTN3* risk variant: C/C, homozygous noncarriers; C/G, heterozygous carriers; and G/G, homozygous carriers. Each value represents a unique sample with the maximum (peak) gene expression value from an individual healthy control or patient. Horizontal bars show mean and SD. ANOVA *P* values adjusted by Tukey’s multiple-comparison test. *P* values are shown only for groups with significant ANOVA *P* < 0.05. Sample numbers for each group are indicated in parentheses.

In a subset of patients (*n* = 298) with available total leukocyte mRNA, we measured autoantigen gene expression of *PRTN3* and *MPO* to address whether the G allele risk variant was associated with elevated autoantigen gene expression. We found that maximum *PRTN3* gene expression in total leukocytes was elevated in G allele carriers compared with noncarriers. In our healthy control cohort (*n* = 130), we observed modest elevated *PRTN3* expression in individuals carrying 1 or 2 copies of the G allele (effect size 0.16, Figure 1A). In the patient cohort (MPO-ANCA and PR3-ANCA, *n* = 298), those with the variant had significantly elevated *PRTN3* gene expression compared with noncarriers (effect size 0.24, Figure 1A). Carriers had higher *PRTN3* gene expression compared with noncarriers (C/G vs. C/C and G/G vs. C/C, *P* = 0.012 and *P* = 0.001, respectively). A similar association was observed using mean expression for patients with multiple samples (Supplemental Figure 2). Patients with MPO-ANCA and PR3-ANCA were included because leukocyte gene expression is not dependent on serotype (12). *MPO* gene expression was higher in patients compared with healthy controls but was not associated with variant genotype (Figure 1B).

*PRTN3* expression was analyzed based on activity status when available (*n* = 190). There was elevated *PRTN3* expression in the homozygous group (G/G) compared with noncarriers (C/C) in active and nonactive groups (*P* = 0.0067 and *P* = 0.047, respectively) (Supplemental Figure 3A). There were no differences in *MPO* gene expression when segregating by activity status (Supplemental Figure 3B). This result suggests the elevated *PRTN3* gene expression among patients with the G allele is not a consequence of overrepresentation of patients with active disease among those carrying the G allele. At the same time, the association of the G allele with *PRTN3* gene expression persists during active disease.

### Genotype association with plasma PR3 levels and PR3-ANCA levels.

**Figure 2:**
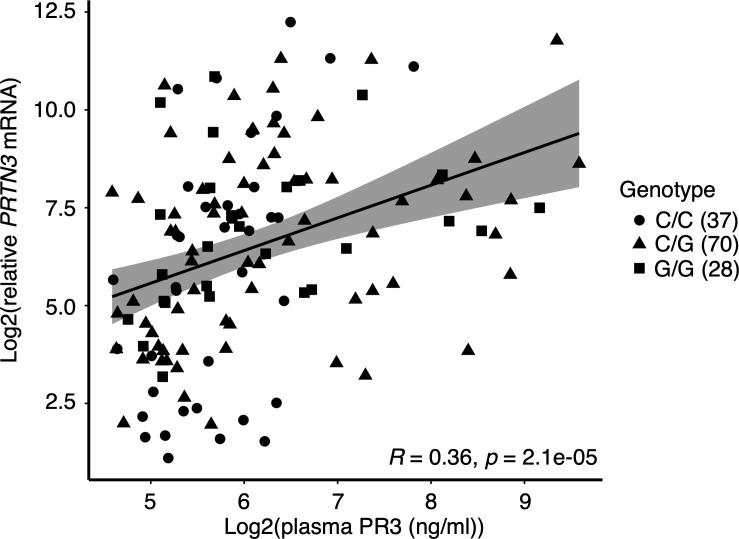
Correlation between leukocyte *PRTN3* gene expression and PR3 plasma levels. Sample numbers for each group are indicated in parentheses.

**Figure 3:**
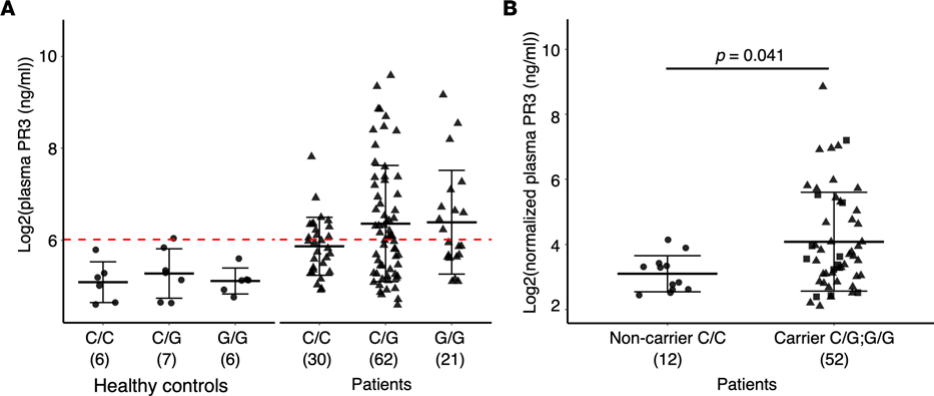
PR3 protein in plasma. (**A**) Circulating PR3 levels in healthy controls (circles) and patients (triangles) by genotype. Red line indicates 2 SDs above mean of healthy controls. (**B**) Plasma PR3 levels normalized to neutrophil count comparing patients who are noncarriers (C/C; circles) versus carriers (C/G; G/G). Squares represent G/G and triangles represent C/G. Horizontal bars indicate mean and SD. Sample numbers are indicated in parentheses under each group. *P* = 0.041 by Wilcoxon’s test.

**Figure 4:**
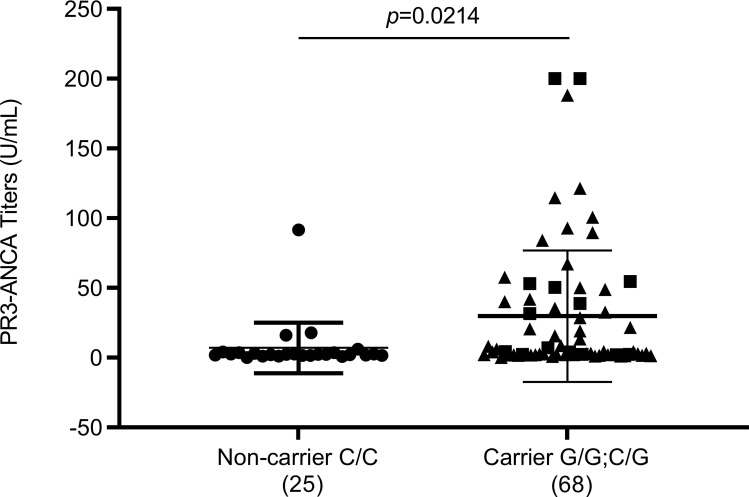
PR3-ANCA levels are higher among carriers compared with noncarriers. *P* = 0.0214 (unpaired *t* test). Squares represent G/G and triangles represent C/G. Sample numbers are indicated in parentheses under each group.

We tested whether elevated leukocyte gene expression, which was associated with the G allele, correlated with increased PR3 protein levels in circulation. PR3 levels were measured in a cohort of patients and healthy individuals with available plasma from the same sample date as leukocyte gene expression data. There was a modest but significant correlation with plasma PR3 and *PRTN3* gene expression (*r* = 0.347, *P* < 0.0005, Figure 2). The healthy controls had low levels of plasma autoantigens and were not different based on genotype (Figure 3A). Patients genotyped as G/G and C/G (carriers) had identical means of plasma PR3 and were combined for further analysis. We normalized plasma PR3 levels to available neutrophil counts. Patients who were variant carriers had higher normalized plasma PR3 compared with noncarriers: *P* = 0.041 (Figure 3B). We used PR3-ANCA titers (autoantibody levels tested clinically) if available at the time of sample to determine whether there was a correlation. We found that among patients with PR3-ANCA, carriers had higher autoantibody titers compared with noncarriers (Figure 4).

### Genotype association with clinical outcomes.

**Figure 5:**
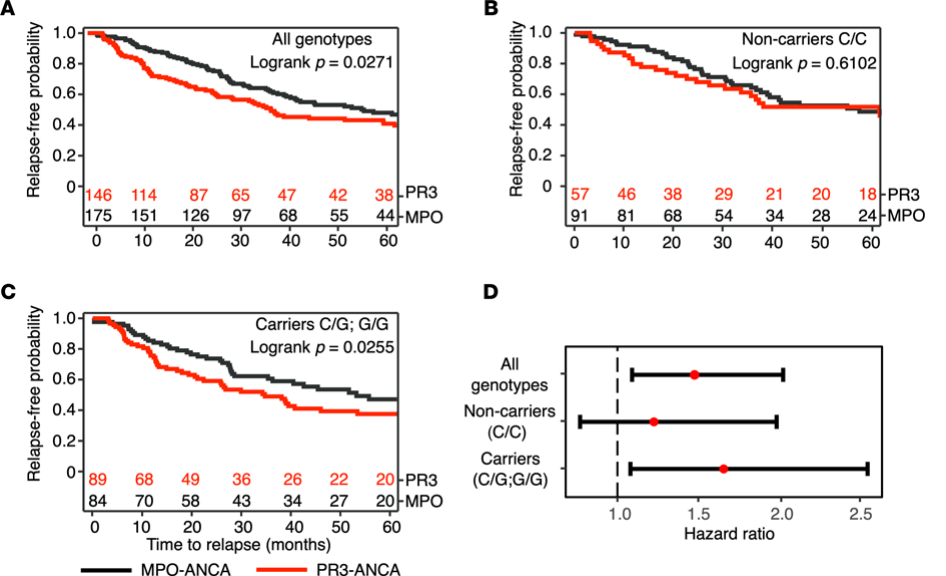
Probability of relapse after achieving remission (*n* = 321). Kaplan-Meier curves stratified according to (**A**) patients with MPO-ANCA (black line) versus PR3-ANCA (red line) in entire cohort (log-rank *P* = 0.027), (**B**) patients with MPO-ANCA versus PR3-ANCA homozygous (C/C) for *PRTN3* nonrisk variant (log-rank *P* = 0.61), and (**C**) patients with MPO-ANCA versus PR3-ANCA heterozygous (C/G) or homozygous (G/G) for the *PRTN3* risk variant (log-rank *P* = 0.025). (**D**) HR comparing relapse risk in PR3- versus MPO-ANCA based on genotype.

To explore the clinical outcomes associated with the molecular findings, we analyzed follow-up patient data (available for 390 of 401 patients). All groups achieved remission at a similar rate (91% C/C, 88% C/G, and 90% G/G, *P* = 0.79). After remission (*n* = 321), PR3 patients relapsed at a higher rate compared with MPO patients (HR 1.48, 95% CI 1.09, 2.02) and had faster time to relapse (log-rank *P* = 0.02, Figure 5A). There was no statistically significant difference in risk of relapse based on variant copy number within the PR3-ANCA group. The frequency of relapse among patients with PR3-ANCA was similar between heterozygotes (C/G, 70.4%) and homozygotes (G/G, 72.2%) (Supplemental Figure 4), so we combined carriers (C/G and G/G) to analyze serotype-specific relapse risk. Among noncarriers (C/C) there was no difference in relapse (HR 1.23, 95% CI 0.77, 1.98) between patients with PR3- or MPO-ANCA (Figure 5B). Carriers with PR3-ANCA had reduced relapse-free survival (Figure 5C) and increased risk of relapse compared with MPO-ANCA (HR 1.66, 95% CI 1.08, 2.54; shown in Figure 5D). Overall, there were no differences in end-stage kidney disease by genotype (data not shown).

## Discussion

**Figure 6:**
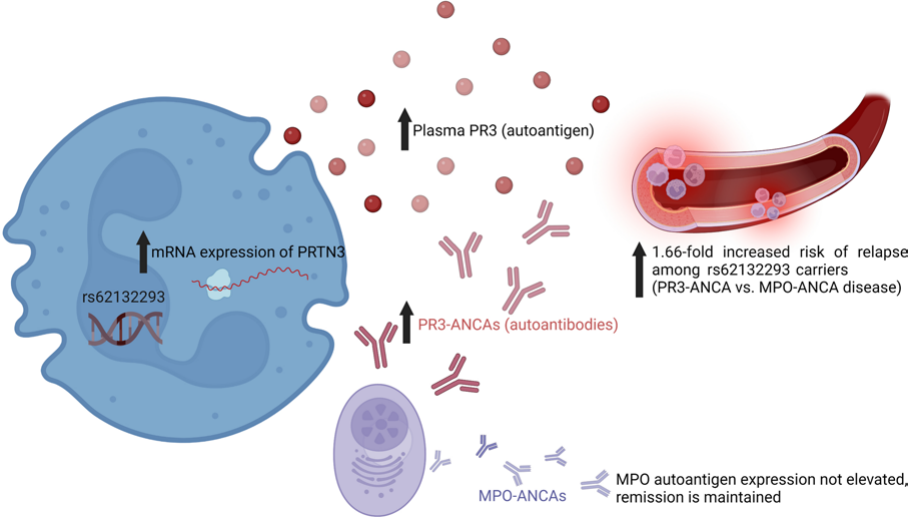
Summary of study findings

Our study is the first to our knowledge to correlate the risk variant, rs62132293, with increased autoantigen gene expression (message) and circulating PR3 in patients with ANCA vasculitis and show the impact on clinical disease phenotype. The *PRTN3* variant had been shown to be an eQTL among healthy volunteers, but this had not been explored in a patient population until now (2). We found higher leukocyte mRNA levels of *PRTN3* in patients inheriting 1 (C/G) or 2 copies (G/G) of the variant. Autoantigen transcript levels have been shown to correlate with protein synthesis of autoantigens in ANCA vasculitis (7, 8, 12, 13). Previous studies have shown that neutrophil gene expression correlates with total leukocyte gene expression, which was used in our current analysis (12). We found a correlation between leukocyte gene expression of *PRTN3* and plasma PR3 levels in our patient cohort. PR3 levels were higher among those with the risk variant, and the difference was significant during active disease (*P* = 0.0496). The association between this risk allele and *PRTN3* gene expression and PR3 protein levels suggests a genetic basis for increased PR3 autoantigen availability. Increased autoantigen expression in neutrophils could lead to enhanced neutrophil activation in the presence of corresponding autoantibodies, explaining the 1.66-fold increased risk of disease relapse among carriers who had PR3-ANCAs compared with MPO-ANCAs (summarized in Figure 6). Among noncarriers, the risk of relapse was similar between PR3-ANCA and MPO-ANCA. Additionally, our results underscore that disease phenotype in ANCA vasculitis may be contingent on autoantigen availability resulting from dysregulated autoantigen gene expression. We have previously shown that an epitope-specific response drives T cell response in MPO- and PR3-ANCA (14, 15). Increased presence of pathogenic autoantigen epitopes may further impact the adaptive response.

Although previous reports suggest coordinated regulation of *MPO* and *PRTN3* expression (16), *MPO* expression did not segregate by *PRTN3* variant genotype, consistent with a cis-regulatory impact on autoantigen gene expression. We postulate that increased availability of PR3 autoantigen is a result of increased transcription of *PRTN3*. The observation that the *PRTN3* variant was associated with elevated *PRTN3* expression and circulating PR3 highlights the importance of autoantigen gene expression in patients with ANCA vasculitis. Increased expression can arise in response to inflammation (17) or disrupted regulatory mechanisms (10). Epigenetic alterations, such as changes in DNA methylation, were associated with *PRTN3* expression and clinical outcomes, and familial ANCA studies support a genetic basis for PR3 expression (18, 19). The data presented here offer additional evidence that elevated *PRTN3* expression in patients with ANCA vasculitis is regulated by mechanisms besides inflammatory response. Importantly, the clinical phenotypes associated with the *PRTN3* variant suggests functional consequences of altered autoantigen gene expression on clinical outcomes in the appropriate context. While we observed the impact of the variant on *PRTN3* expression among all patients with ANCA, the clinical impact of the variant on relapse was restricted to patients with PR3-ANCA.

Our study focused on 1 particular genetic variant, though it should be noted that rs62132293 is in strong linkage disequilibrium (*R*^2^ > 0.74) with other variants associated with *PRTN3* gene expression (Supplemental Figure 5) and potentially represents an allelic signal contributing to *PRTN3* gene expression. Other variants that colocalize with rs62132293 include the ANCA GWAS variant from a study of European individuals (rs62132295, *r*^2^ = 0.94) and the variant most strongly associated with *PRTN3* expression (rs138303849, *r*^2^ = 0.75) as reported by the Genotype-Tissue Expression (GTEx) project (20). The rs62132293 variant is also in linkage disequilibrium with a variant associated with plasma PR3 protein levels (rs7254911, *r*^2^ = 0.77) (5). Other variants were not specifically tested in our population, though we speculate that the rs62132293 variant identifies a cis-regulatory element that impacts *PRTN3* gene expression and PR3 plasma levels. Previous studies identified epigenetic mechanisms regulating autoantigen gene expression that were disrupted in patients and marked a transcriptionally permissive state (10, 18, 21). We propose that the chromatin state at *PRTN3* works in concert with the underlying genetic sequence to orchestrate *PRTN3* expression. Future investigations will test this model and identify the contribution of genetic variants to expression.

Despite using a large cohort with extensive follow-up, our study has several limitations. Patients homozygous for the G allele made up only 10% of our cohort, limiting subgroup data analysis. Although autoantigen expression is generally higher in active disease, activity status alone does not explain the association of genotype with *PRTN3* expression; paired sample data are needed to unequivocally address the impact of activity status. Risk of relapse within the PR3-ANCA cohort was not based on variant carrier status. Possible explanations include other clinical differences in patients with PR3-ANCA or other genetic influences, such as HLA variants, that predispose patients with PR3-ANCA to likelihood of relapse (15).

We believe this study is the first to report higher leukocyte autoantigen expression and plasma PR3 in patients with rs62132293 (*PRTN3*) variant and increased relapse among carriers who have PR3-ANCAs compared with those with MPO-ANCAs. This variant may not solely explain the clinical severity of PR3-ANCA disease, though it certainly the role of autoantigen expression. While the current clinical focus is on presence of autoantibodies and symptoms, our data warrant further investigation into threshold autoantigen values that could offer relapse prediction among patients with PR3-ANCA vasculitis.

## Methods

### Study cohort.

Patients with vasculitis (prevalent and newly diagnosed) at the University of North Carolina at Chapel Hill clinics and healthy volunteers were screened and enrolled in the Glomerular Disease Collaborative Network, and patients were classified according to the Chapel Hill Consensus Conference definitions (22).

Patients were followed prospectively and clinicians reviewed charts to confirm disease activity, relapse, and remission. Disease was defined by the 2003 Birmingham Vasculitis Activity Score (BVAS) and clinical activity (23). Disease onset was defined as onset of clinical symptoms and/or histopathological diagnosis. Remission was defined by BVAS of 0 and no clinical or laboratory evidence of active disease. Active disease was defined as BVAS > 0 with clinical and/or laboratory evidence of disease. For evaluation of relapse, complete remission after initial diagnosis (on or off treatment) was required. Relapse was defined by BVAS and/or recurrent clinically active disease and the need for new or escalation of immunosuppressive therapy. ANCA serotypes were assessed by indirect immunofluorescence (IF) with positive C-ANCA or P-ANCA and antigen-specific PR3 and MPO ELISA (24). MPO and/or P-ANCA were classified together, and PR3 and/or C-ANCA were classified together. Patients with only P-ANCA prior to MPO availability had negative anti-neutrophil antibodies. Patients were considered seronegative if they had clinicopathologic features of ANCA-associated vasculitis but were ANCA negative by IF and ELISA. Patients with suspected or confirmed drug-induced forms of ANCA vasculitis or overlapping diseases were excluded.

### Variant genotyping.

All included patients had available DNA samples. Affymetrix Axiom Biobank Genotyping Array was used for genotyping 286 patients as part of the Vasculitis Clinical Research Consortium GWAS (2). An additional 115 patients and 130 healthy controls were genotyped by PCR (including confirmation of 15 of the GWAS patients), via amplification of the SNP-containing region using forward primer 5′ GAGCTGACTCATGGCTGAAACCAAC 3′, reverse primer 5′ TGATGTGTATTAAAGAACTAGAGCT 3′. PCR products were separated by agarose gel electrophoresis and imaged using iBright FL1000 (Invitrogen, Thermo Fisher Scientific) (Supplemental Figure 1).

### Leukocyte PRTN3 mRNA quantitative PCR.

Total leukocytes from patients and healthy controls were used for RNA isolation. When possible, “active” samples were obtained at onset; otherwise, samples during therapy or remission were obtained. RNA was isolated from leukocytes using the AllPrep DNA/RNA Mini Kit (QIAGEN). Quantitative detection of *PRTN3* and *MPO* mRNA was performed with TaqMan RNA-to-C_T_ 1-Step Kit (Applied Biosystems, Thermo Fisher Scientific). Quantitative reverse transcription PCR was performed on an ABI PRISM 7900HT (Applied Biosystems, Thermo Fisher Scientific) using primer and probe assays listed (Supplemental Table 1). Quantitative detection of *PRTN3* and *MPO* mRNA levels was determined by 2-ΔΔCt calculations as described previously (21). The maximum gene expression was used for each patient with multiple samples measuring gene expression (or the mean expression for patients with multiple samples).

### Quantification of plasma PR3.

Plasma levels of circulating PR3 were measured by ELISA (Eagle Biosciences) using available frozen plasma samples from the same date as the maximum gene expression data. Assays were performed according to the manufacturer’s instructions and included negative controls. Each sample was tested in duplicate. ELISA data were analyzed using GraphPad Prism with an appropriate curve-fitting equation. The plasma PR3 levels were normalized to neutrophil counts for comparison. Neutrophil counts were derived from clinical testing within 2 weeks of sample.

### Statistics.

Categorical measures were reported as number and percentage, and continuous measures as mean and SD or median and IQR, if not normally distributed. Variables by genotype status were compared using Fisher’s exact tests for categorical variables and Kruskal-Wallis tests for continuous variables. Kaplan-Meier estimates and log-rank test were used to assess differences in time to relapse of various genotypes in all patients and by ANCA serotypes separately. Multivariable Cox proportional hazards models for time to relapse were used with HRs, *P* values, and 95% CIs reported. Gene expression data were transformed using log_2_, the analysis was done by 1-way ANOVA, and *P* values were adjusted by Tukey’s multiple-comparison test. Age between genotype groups was compared using Wilcoxon’s 2-sample test and adjusted by stepdown Bonferroni’s. Plasma protein levels were also compared using Wilcoxon’s 2-sample test. The Cochran-Mantel-Haenszel method was used to evaluate associations between ANCA specificity (MPO/P, PR3/C) and an ordinal measure of genotype controlling for disease relapse. Figure 4 used unpaired, 2-tailed *t* test. Statistical significance was defined as *P* < 0.05. Statistical analyses were done and figures were made using SAS (Version 9.4, SAS Institute), GraphPad Prism (Version 6.04 for Windows, GraphPad Software), and R (Version 4.0.2, R Core Team) using tidyverse and ggplot2 packages (https://www.tidyverse.org).

### Study approval.

Participants provided informed, written consent and study procedures were in accordance with guidelines of the IRB (study no. 97-0523; study title: Identification of Causes and Markers of Renal Disease) of the University of North Carolina at Chapel Hill Office of Human Research Ethics. The IRB approved the study (Reference ID 381078).

## Author contributions

DPC and DJC designed research, conducted experiments, analyzed data, prepared figures, and wrote the manuscript. DPC, VKD, EYW, and RJF chart reviewed patients. DJC and JY conducted mRNA gene expression experiments. CPA, DM, TS, DPC, and DJC conducted the genotyping experiments. DPC and DJC conducted PR3 ELISAs. YH and SLH performed and interpreted statistical analyses. JCJ helped with manuscript revision. RJF helped with study design and critically read the manuscript. All authors reviewed and approved the final version of the manuscript.

## Supplementary Material

Supplemental data

## Figures and Tables

**Table 1:**
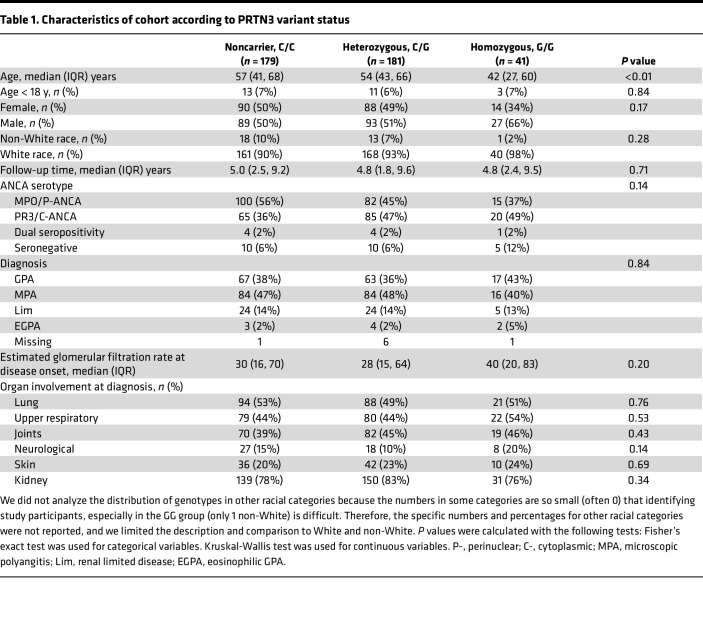
Characteristics of cohort according to PRTN3 variant status

## Version 1 01/10/2023

In-Press Preview

## Version 2 02/22/2023

Electronic publication
