# Supplementary material for: PRTN3 variant correlates with increased autoantigen levels and relapse risk in PR3-ANCA versus MPO-ANCA disease
Source: JCI Insight. 2023 Feb 22;8(4):e166107. doi: 10.1172/jci.insight.166107 (PMC9977496; doi:10.1172/jci.insight.166107)
Supplement: Supplemental data [file jciinsight-8-166107-s189.pdf]

List of Supplementary Figures and Tables:

Supplemental Figure 1. Example of agarose gel visualization of genotyping of samples
Supplemental Figure 2. Mean *PRTN3* expression by variant.
Supplemental Figure 3: Autoantigen gene expression in leukocytes based on variant carrier status and activity status.
Supplemental Figure 4. Relapse or remission status by MPO and PR3 after achieving remission within each genotype group.
Supplemental Figure 5. Map of *PRTN3* associated variants in linkage disequilibrium with rs62132293.
Supplemental Table 1. List of primers and probes for TaqMan qPCR.
Supplemental Table 2. Baseline Characteristics of cohort based on variant status, PR3/c-ANCA patients.
Supplemental Table 3. Baseline Characteristics of cohort based on variant status, MPO/p-ANCA patients.

Supplementary Figure 1. Example of agarose gel visualization for genotyping samples. For PCR, genomic DNA was isolated from whole blood patient samples and the region upstream of *PRTN3* containing the SNP was amplified using: forward primer 5'-GAGCTGACTCATGGCTGAAACCAAC-3', reverse primer 5'-TGATGTGTATTAAAGAACTAGAGCT-3'. PCR products were separated by agarose gel electrophoresis and imaged using iBrightFL1000(Invitrogen). Genotype determination was by visualization as the G allele is associated with a deletion within this region resulting in a smaller band compared to C allele.

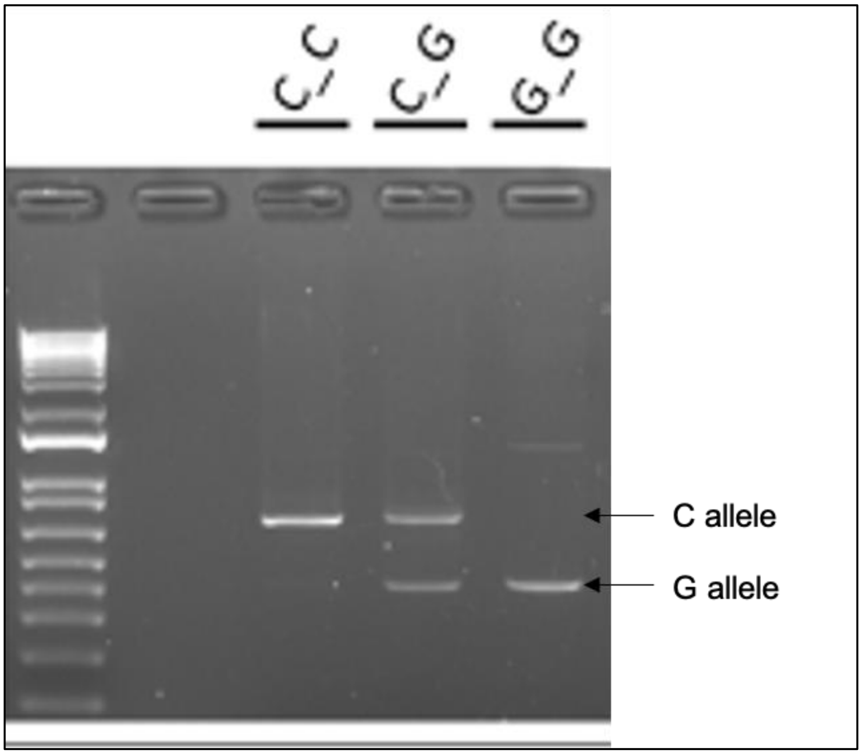

Supplementary Figure 2. Mean PRTN3 expression by variant.

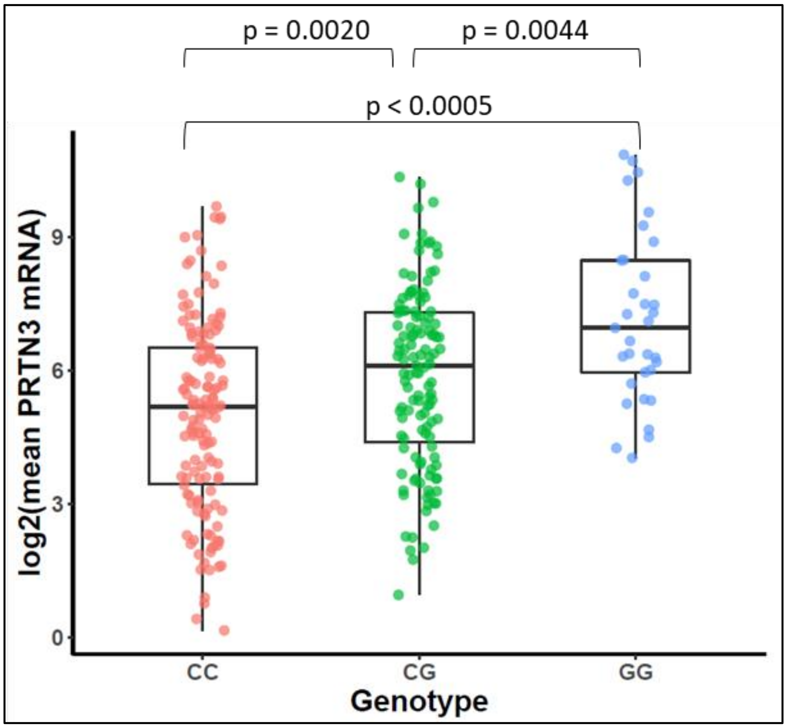

Supplementary Figure 3: Autoantigen gene expression in leukocytes based on variant carrier status and activity status. A. Autoantigen gene expression of *PRTN3* in leukocytes remains elevated in homozygous groups regardless of activity status. B. Autoantigen gene expression of MPO does not change based on activity status. If multiple samples were available from individual patient, sample with maximum expression (peak) was used. Patient disease status was classified as active or remission according to criteria described in Methods. ANCA serotypes were determined by indirect immunofluorescence and antigen-specific PR3 and MPO ELISA. Mean and standard deviation are shown, ANOVA P values adjusted by Tukey's multiple comparison test.

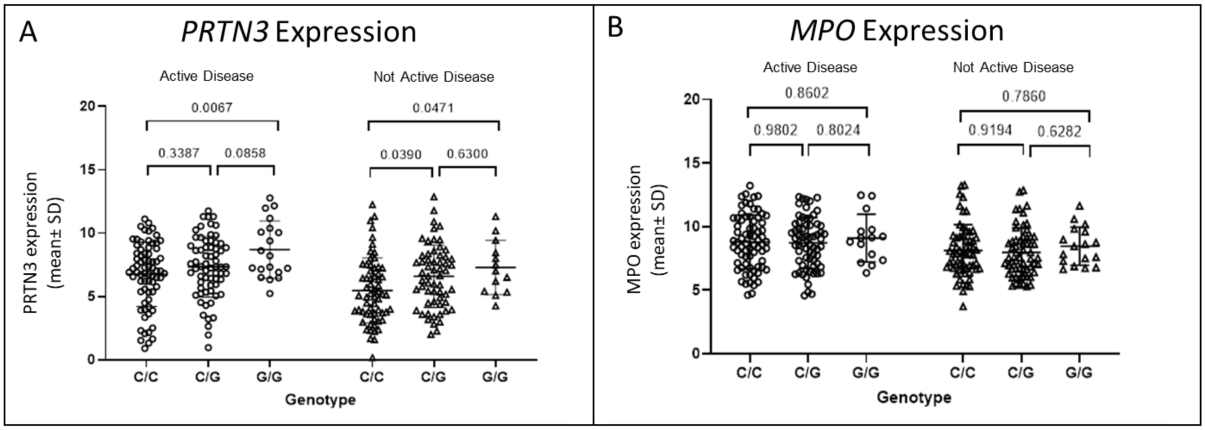

Supplementary Figure 4. Relapse or remission status by MPO and PR3 after achieving remission within each genotype group.

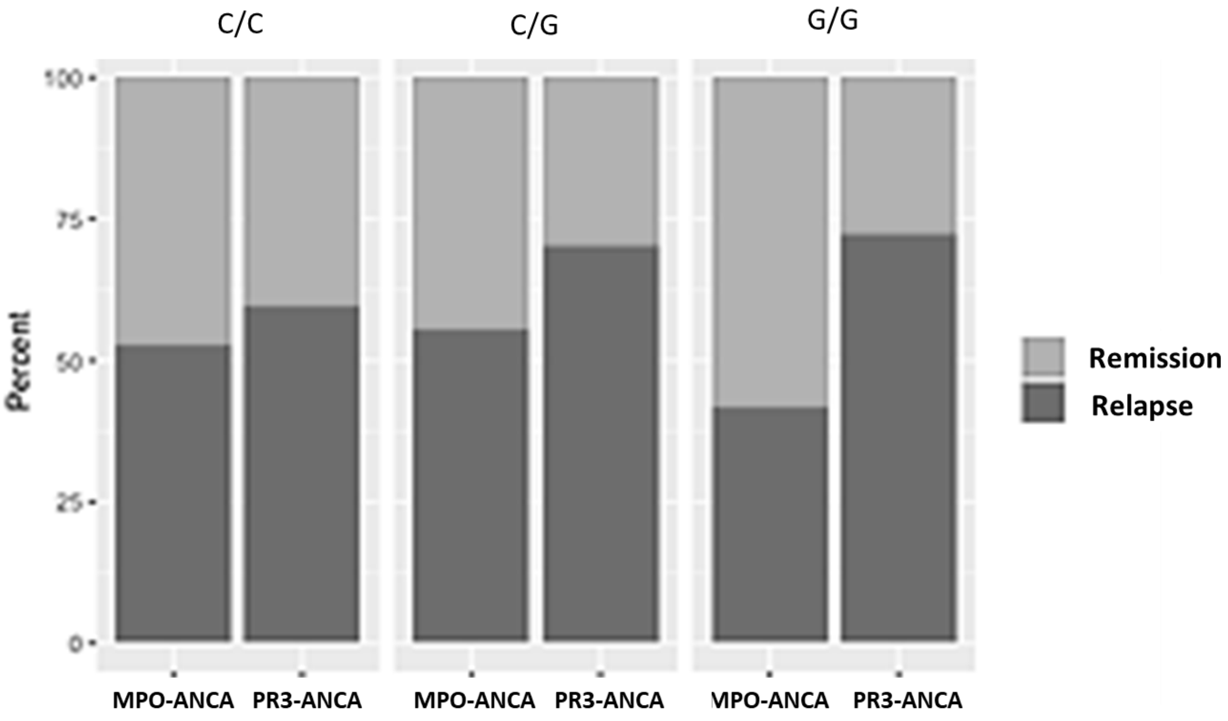

	**MPO, C/C**	**PR3, C/C**	**MPO, C/G**	**PR3, C/G**	**MPO, G/G**	**PR3, G/G**
**Remission (n,%)**	43(47.2%)	23(40.3%)	32(44.4%)	21(29.6%)	7(58.3%)	5(27.8%)
**Relapse (n, %)**	48(52.8%)	34(59.7%)	40(55.6%)	50(70.2%)	5(41.7%)	13(72.2%)

Supplementary Figure 5. The rs62132293 variant is in strong linkage disequilibrium with other variants associated with PRTN3 gene expression in whole blood, including the ANCA GWAS variant from a study of European individuals (rs62132295, r2=.94) and the variant most strongly associated with PRTN3 (rs138303849, r2=.75) as reported by the Genotype-Tissue Expression (GTEx) project. The GWAS variant is also in linkage disequilibrium with a variant associated with serum PR3 (PRTN3) protein levels (rs7251804, r2=.77).

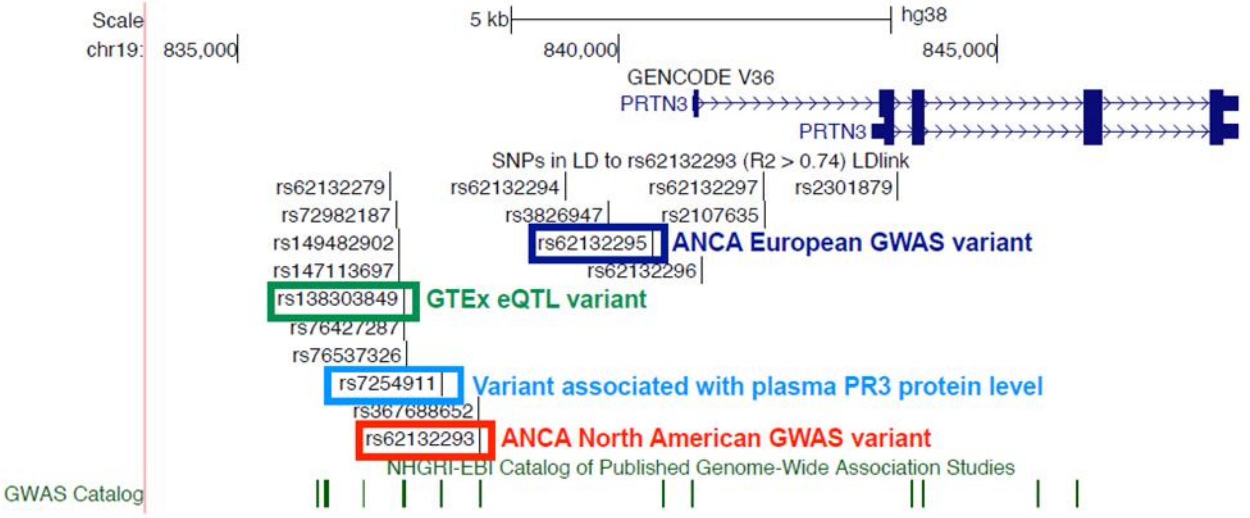

Supplemental Table 1

Primers and Probe for TaqMan quantitative real-time PCR			
Gene	Primer	Sequence	Company
MPO	Forward	5'-CCA GGA AGC CCG GAA GAT-3'	Integrated DNA Technologies, INC Coralville, IA
	Reverse	5'-CGG AAG GCA TTG GTG AAG A-3'	
	Probe	FAM-TGC CCA CGT ACC GTT CCT ACA ATG ACT C-TAMRA	
PRTN3	Forward	5'-TGT CAC CGT GGT CAC CTT CTT-3'	
	Reverse	5'-CCC CAG ATC ACG AAG GAG TCT AT-3'	
	Probe	FAM-TTG CAC TTT CGT CCC TCG CCG-TAMRA	
COX5B	Forward	5’-TGG CAT CTG GAG GTG TT-3'	
	Reverse	5'-GTC CAG TCC CTT CTT TGC AGC-3'	
	Probe	FAM-TGA TGA AGA GCA GGC GAC TGG GTT G-MGB	
Gene	TaqMan Gene Expression Assay		Company
MPO	Hs00924296_m1		ThermoFisher, Waltham, MA
PRTN3	Hs01597752_m1		
COX5B	HS00426948_m1		

Supplemental Table 2: Baseline Characteristics of cohort according to SNP status, PR3/C patients

Variables	CC (n=65)	CG(n=85)	GG(n=20)	p-value
**Age, median(IQR) Years**	52.97(39.12, 61.67)	52.77(41.23, 65.00)	32.67(24.07, 45.26)	0.0037
**Age<18yo**	5(7.69%)	5(5.88%)	2(10.00%)	0.6948
**Male**	36(55.38%)	52(61.18%)	15(75%)	0.2818
**Race (non-white)**	5(7.69%)	4(4.71%)	0(0.00%)	0.4976
**Follow-up time median (IQR) Years**	5.42(2.16, 10.61)	5.21(1.64, 9.24)	7.31(3.18, 11.74)	0.3414
**Diagnosis**				0.5181
**Missing**	1	2	1	
**EGPA**	1(1.56%)	0(0.00%)	1(5.26%)	
**GPA**	35(54.69%)	43(51.81%)	12(63.16%)	
**Lim**	3(4.69%)	5(6.02%)	0(0.00%)	
**MPA**	25(39.06%)	35(42.17%)	6(31.58%)	
**Estimated GFR at enrollment, median(IQR) (based on peak entry Cr)**	33.50(20.00, 84.82)	26.15(13.65, 62.42)	56.93(23.36, 85.45)	0.0499
**Highest BVAS, median(IQR)**	6.00(0,13.00)	8.50(0,16.00)	11.00(6.00, 13.00)	0.4258
**BVAS (Max PR3) median(IQR)**	1.00(0.00, 7.00)	0.00(0.00, 6.00)	1.50(0.00, 10.00)	0.9563
**Organ Involvement , n (%)**				
**Lung**	43(66.15%)	57(67.06%)	12(60%)	0.827
**Upper Resp**	40(61.54%)	48(56.47%)	14(70%)	0.5258
**Joints**	33(50.77%)	44(51.76%)	13(65%)	0.5396
**Neurological**	12(18.46%)	11(12.94%)	6(30%)	0.1629
**Skin**	18(27.69%)	22(25.88%)	7(35%)	0.6937
**Kidney**	49(75.38%)	74(87.06%)	14(70%)	0.0808

Supplemental Table 3: Baseline Characteristics of cohort according to SNP status, MPO/P patients

Variables	CC (n=100)	CG(n=82)	GG(n=15)	p-value
**Age, median(IQR) Years**	58.57(42.85, 70.00)	54.01(45.30, 68.71)	48.53(23.87, 69.87)	0.4688
**Age<18yo**	7(7.00%)	5(6.10%)	1(6.67%)	1.0000
**Male**	46(46%)	36(43.9%)	9(60%)	0.5345
**Race (non-white)**	12(12%)	7(8.54%)	1(6.67%)	0.7636
**Follow-up time median (IQR) Years**	4.60(2.68, 9.26)	4.84(2.19, 9.87)	4.26(2.08, 7.41)	0.8618
**Diagnosis**				0.8224
**Missing**	0	3	0	
**EGPA**	2(2%)	3(3.80%)	1(6.67%)	
**GPA**	24(24%)	16(20.25%)	2(13.33%)	
**Lim**	20(20%)	17(21.52%)	4(26.67%)	
**MPA**	54(54%)	43(54.43%)	8(53.33%)	
**Estimated GFR at enrollment, median(IQR) (based on peak entry Cr)**	25.07(14.82, 45.20)	27.67(16.40, 63.58)	24.39(11.57, 56.01)	0.7613
**Highest BVAS, median(IQR)**	7.00(2.00,12.00)	7.00(3.00, 14.00)	5.00(3.00, 17.00)	0.9837
BVAS Max PR, Median(IQR)	0.00(0.00, 6.00)	2.00(0.00, 6.00)	4.00(0.00, 6.00)	0.4703
**Organ Involvement , n (%)**				
**Lung**	44(44%)	27(32.93%)	5(33.33%)	0.3022
**Upper Resp**	33(33%)	27(32.93%)	5(33.33%)	1.0000
**Joints**	32(32%)	33(40.24%)	6(40%)	0.4785
**Neurological**	15(15%)	7(8.54%)	1(6.67%)	0.3810
**Skin**	14(14%)	15(18.29%)	2(13.33%)	0.7917
**Kidney**	81(81%)	67(81.71%)	13(86.67%)	0.9608
